# Supplementary material for: Gut barrier defects, intestinal immune hyperactivation and enhanced lipid catabolism drive lethality in NGLY1-deficient Drosophila
Source: Nat Commun. 2023 Sep 13;14:5667. doi: 10.1038/s41467-023-40910-w (PMC10499810; doi:10.1038/s41467-023-40910-w)
Supplement: Supplementary file 1 — Supplementary Information [file 41467_2023_40910_MOESM1_ESM.pdf]

Gut barrier defects, intestinal immune hyperactivation and enhanced lipid catabolism drive lethality in NGLY1-deficient *Drosophila*

Ashutosh Pandey, Antonio Galeone, Seung Yeop Han, Benjamin A Story, Gaia Consonni, William F Mueller, Lars M Steinmetz, Thomas Vaccari & Hamed Jafar-Nejad

Supplementary Data

Supplementary Figures 1-5

Supplementary Tables 1-2

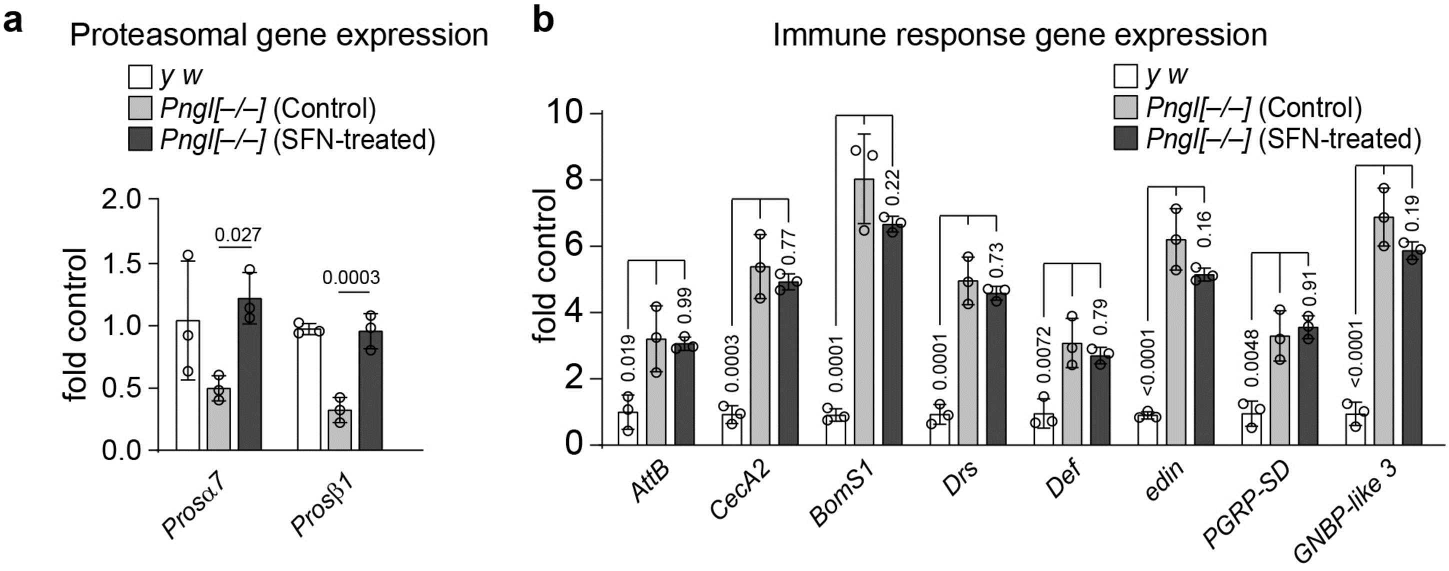

Supplementary Figure 1. Related to Figure 1

Hyperactivation of immune genes in *Pngl* mutants cannot be explained by NFE2L1 associated proteasomal inhibition. a Graph showing expression of proteasomal genes in the indicated genotypes. **b** Graph showing expression of immune genes in the indicated genotypes. In both panels, mean ± standard deviation of three independent replicates is shown. Numbers on the bars indicate the *P* values. Significance is ascribed as $P<0.05$ using one-way ANOVA with multiple comparisons followed by Fisher's LSD in (a) and Šidák correction in (b). Source data are provided as Source Data file.

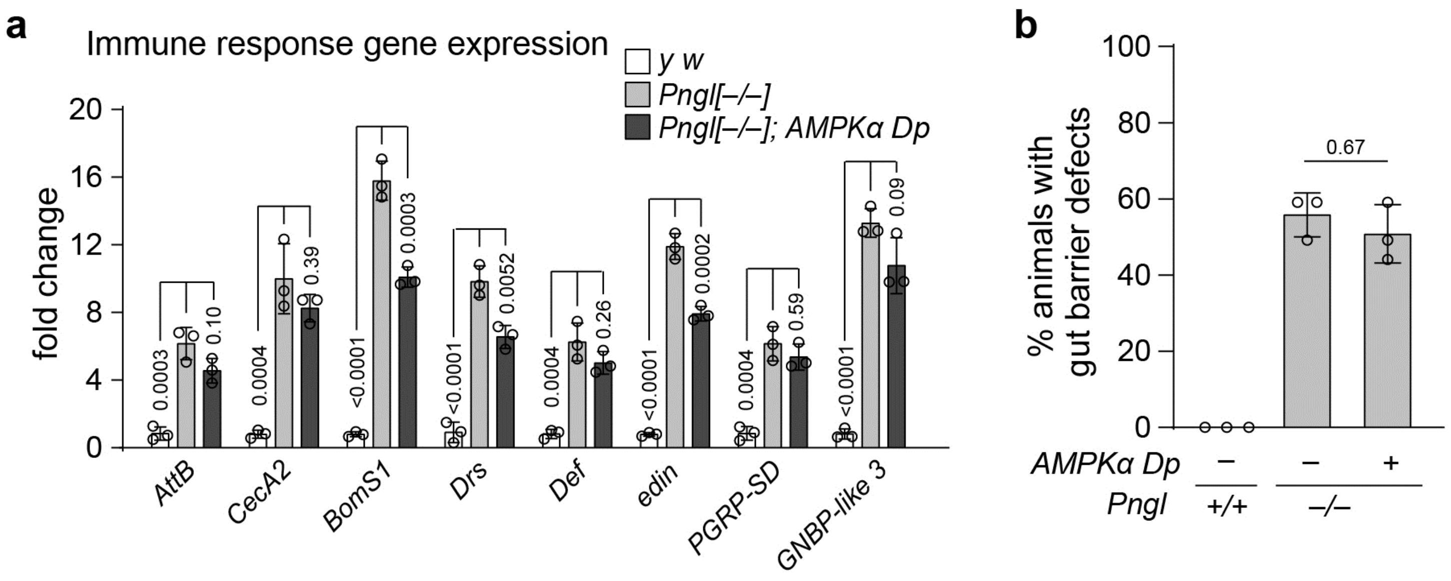

Supplementary Figure 2. Related to Figure 1 and Figure 4

Innate immune gene activation and gut barrier defect in *Pngl* mutants cannot be explained by decreased *AMPKα* level. a Graph showing innate immune gene expression in the indicated genotypes. **b** Graph showing quantification of the gut barrier defect phenotype in indicated genotypes. In both panels, mean ± standard deviation of three independent replicates is shown. Numbers on the bars indicate the *P* values. Significance is ascribed as *P* value <0.05 using one-way ANOVA with multiple comparisons followed by Šidák correction. Source data are provided as Source Data file.

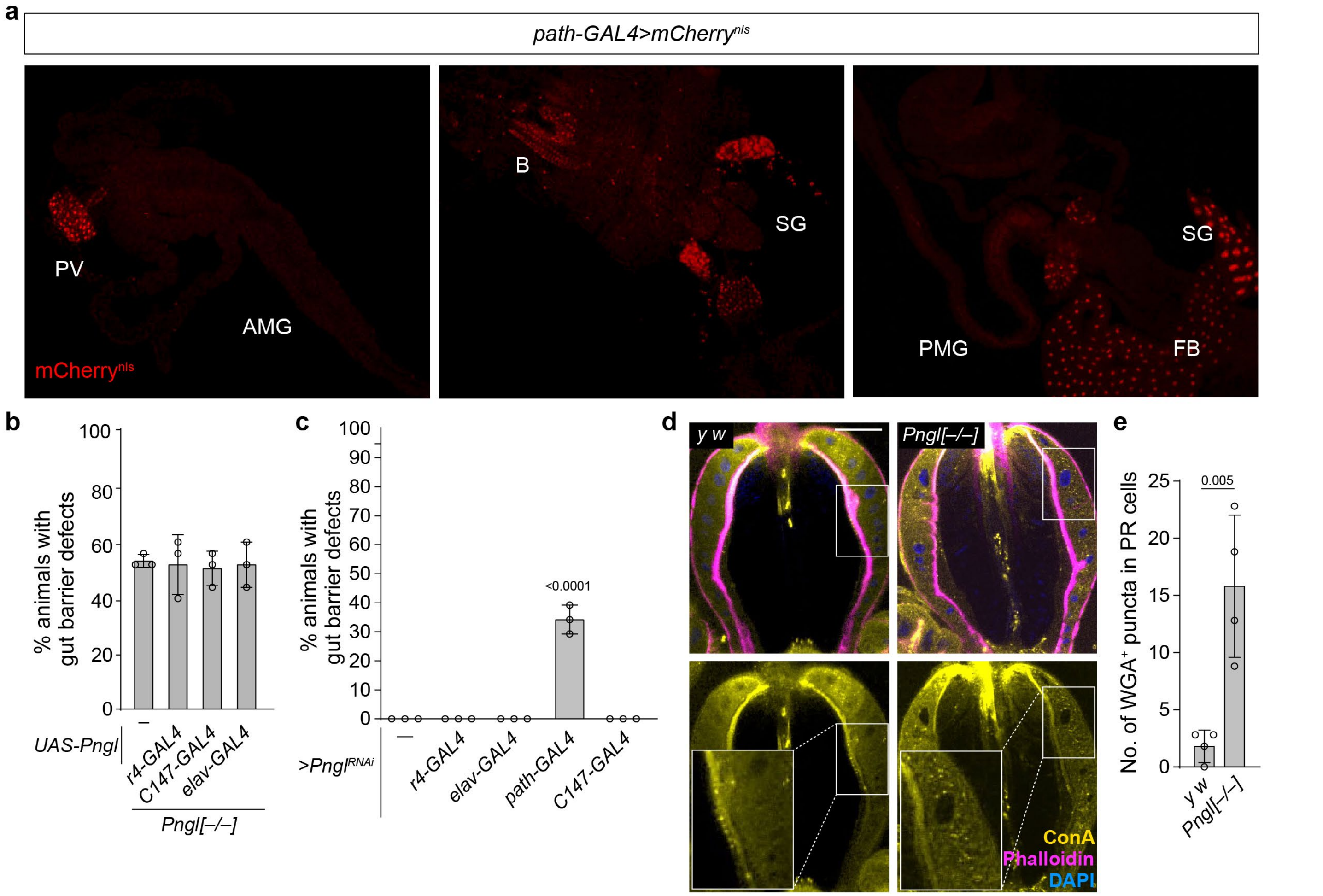

Supplementary Figure 3 Related to Figure 4

Loss of *Pngl* in PR cells results in gut barrier defects. a *Path-GAL4* expression pattern using *UAS-mCherry*^{nls}. B, Brain; AMG, Anterior midgut; PMG, Posterior midgut; SG, Salivary gland; FB, Fat body; PV, Proventriculus. **b** Graph showing % animals of indicated genotypes with gut barrier defects (n=3 independent replicates). *C147-GAL4* and e*lav-GAL4* drive expression in salivary glands and neurons, respectively. **c** Graph showing % animals with gut barrier defect upon *Pngl* knockdown driven by indicated GAL drivers (n=3 independent replicates). Only *path-GAL4* driven *Pngl* knockdown shows gut barrier defects. **d** Confocal images showing DAPI, phalloidin, and WGA lectin in the proventriculus region of control (*y w*) and *Pngl* mutant larvae. Five animals were scored for each group. Scale bar is 50 µm. **e** Graph showing the quantification of WGA^{+} puncta number in control and *Pngl* mutants (n=4 independent replicates). In all panels, mean ± standard deviation is shown. *P* values are indicated on the bars. Significance is ascribed as *P*<0.05 using one-way ANOVA followed by Šidák correction in (b, c) and a two-tailed unpaired t-test in (e). Source data are provided as Source Data file.

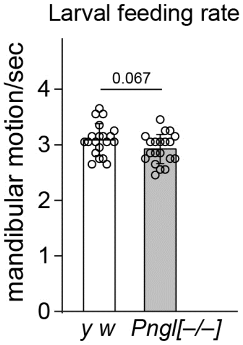

Supplementary Figure 4. Related to Figure 6

***Pngl* mutants exhibit feeding behavior comparable to wild-type.** Graph showing larval feeding behavior in wild-type and *Pngl* mutants. Each circle represents a larva (n=20 for each genotype). Mean ± standard deviation is shown. The number on the bar indicates *P* value. Significance is ascribed as *P*<0.05 using two-tailed unpaired student's t-test.

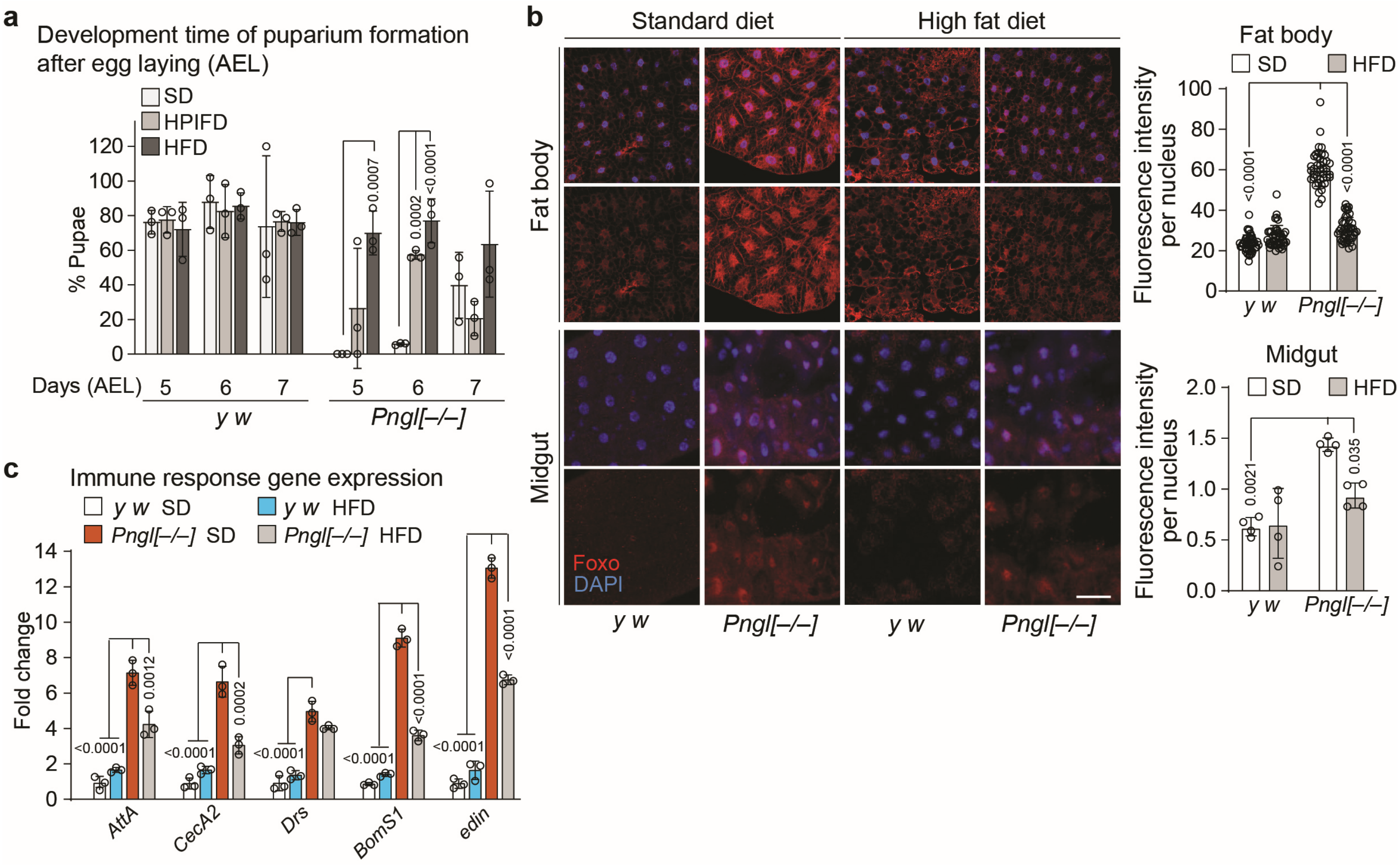

Supplementary Figure 5. Related to Figure 7

HFD rescues the developmental delay and improves lethality rescue in *Pngl* mutants. a Graph showing developmental time of puparium formation after egg laying (AEL) in control and *Pngl* mutants with the indicated diets (n=3 independent replicates). SD, standard diet; HPIFD, high-protein intermediate-fat diet; HFD, high-fat diet. **b** Confocal images showing DAPI (blue) and Foxo staining (red) and quantification of Foxo nuclear localization in the midgut (n=42-45 nuclei from three animals) and fat body (n=4 animals) of wild-type and *Pngl* mutants with the indicated diets. Scale bar is 50 µm. **c** Graph showing innate immune gene expression in control and *Pngl* mutants with or without HFD feeding (n=3 independent replicates). In all panels, mean ± standard deviation is shown. Numbers on the bars indicate the *P* values. Significance is ascribed as *P*<0.05 using two-way ANOVA with multiple comparisons followed by Šidák correction. Source data are provided as Source Data file.

Supplementary Table 1. Related to Figure 7

Detailed composition of the standard diet (SD), high-protein, intermediate-fat diet (HPIFD), and high-fat diet (HFD) used in the study.

**Standard diet (SD)**					
Ingredients	Weight (g)	Calorie (kcal)	Fats (g)	Carbohydrates (g)	Proteins (g)
Water	1000	0	0	0	0
Yeast	25	97.5	1.5	8.25	12.5
Agar	7	0	0	0	0
Corn flour	93	321.78	0.65	72.54	6.04
Molasses	155	444	0	118.4	0
Propionic acid	7	0	0	0	0
Tegosept	20	0	0	0	0

**High-protein, intermediate-fat diet (HPIFD)**					
Ingredients	Weight (g)	Calorie (kcal)	Fats (g)	Carbohydrates (g)	Proteins (g)
Water	1000	0	0	0	0
Yeast	19.23	75	1.15	6.35	9.62
Agar	7	0	0	0	0
Corn flour	19.23	67.31	0.14	15.15	1.26
Sucrose	32.3	129.23	0	32.3	0
Propionic acid	7	0	0	0	0
Tegosept	20	0	0	0	0
Clarified butter	25.92	232.77	25.85	0	0
Soy protein	69.6	258.31	1.04	0	62.65

**High-fat diet (HFD)**					
Ingredients	Weight (g)	Calorie (kcal)	Fats (g)	Carbohydrates (g)	Proteins (g)
Water	1000	0	0	0	00
Yeast	32.76	128.27	1.95	10.85	16.44
Agar	7	0	0	0	0
Corn flour	15.12	52.1	0.11	11.72	0.98
Sucrose	25.2	119.2	0	25.07	0
Propionic acid	7	0	0	0	0
Tegosept	20	0	0	0	0
Clarified butter	50.4	452.58	50.3	0	0

Supplementary Table 2. List of the primer sequences used in the gene expression analysis in this study.

Gene	Primer (5'-3')
AttA	Forward- CTCCTGCTGGAAAACATC<br>Reverse- GCTCGTTTGGATCTGACC
AttB	Forward- GGGTAATATTTAACCGAAGT<br>Reverse-GTGCTAATCTCTGGTCATC
CecA1	Forward- CATTGGACAATCGGAAGCTGGGTG<br>Reverse- TAATCATCGTGGTCAACCTCGGGC
CecA2	Forward- ATTAGATAGTCATCGTGGTT<br>Reverse- GTGTTGGTCAGCACACT
Def	Forward- GTTCTTCGTTCTCGTGG<br>Reverse- CTTTGAACCCCTTGGC
Drs	Forward- AGTACTTGTTCGCCCTCTTGGCTG<br>Reverse- CCTTGTATCTTCCGGACAGGCAGT
Edin	Forward- AGTTCCAGACCAGTCCAGAG<br>Reverse- CGACCCACTTGGTTGTCCTT
BomS1	Forward- CTCGGTCTGCTGGCTGTGGC<br>Reverse- CCGTGGACATTGCACACCC
Dso2	Forward- CTGTCTGAAGATCTGCGGCT<br>Reverse- TTGAATCAACGTGTGTCCGC
PGRP-SD	Forward- ACTTGGATCGGTTTGCTCATC<br>Reverse- AGGGAGTTTCCATGCTGTCTAT
PGRP-LA	Forward- CTAAGGTGACCAGAAGCCCG<br>Reverse- CGGCCAGTTCCGGATTTCTT
PGRP-SB1	Forward- CCGCAATTTCAGCGATATTGG<br>Reverse- GGGAGTTGATCCCTGGAG
GNBP-like 3	Forward- GTCAAGGTCAACTCACCGAAG<br>Reverse- CGTGAAAAGCGAATAGGGAAATG
Lip3	Forward- AAAACGGGTGAATCTTCCAACC<br>Reverse- CCAGCATATAGGCCAGGGA
CG6271	Forward- ATGGATGTCGCTGAGGAGTG<br>Reverse- TGAAGAGGTAGAAGTTCACGGG
CG6277	Forward- TTGCCGAACAGTGGATGGAAG<br>Reverse- AAAGGTAGAACTTTACGGGAACG
CG8093	Forward- CACCATTGTTAGGGGACACGG<br>Reverse- GTGCATTGTGAGGATGTAACCA
InR	Forward- AAGCGTGGGAAAATTAAGATGGA<br>Reverse-GGCTGTCAACTGCTTCTACTG
Actin5C	Forward- TTGTCTGGGCAAGAGGATCAG<br>Reverse- ACCACTCGCACTTGCACTTTC
