# Supplementary material for: Gut barrier defects, intestinal immune hyperactivation and enhanced lipid catabolism drive lethality in NGLY1-deficient Drosophila
Source: Nat Commun. 2023 Sep 13;14:5667. doi: 10.1038/s41467-023-40910-w (PMC10499810; doi:10.1038/s41467-023-40910-w)
Supplement: Supplementary file 3 — Description of Additional Supplementary Files [file 41467_2023_40910_MOESM3_ESM.pdf]

Description of Additional Supplementary Files

File Name: Supplementary Data 1
Description: **Related to Figure 1. RNA-seq pairwise comparisons.** The full dataset for each of the three pairwise comparisons involving *Pngl*$^{-/-}$ midguts is provided as an Excel file (Pngl$^{-/-}$ vs y w, Pngl$^{-/-}$ vs Pngl$^{+/-}$, and Pngl$^{-/-}$ vs Pngl$^{-/-}$; Pngl Dp/+). FC, fold change; CPM, counts per million; LR, likelihood ratio; FDR, false discovery rate.

File Name: Supplementary Data 2
Description: **Related to Figure 1. List of downregulated and upregulated genes in all categories shown in the Venn diagrams in Figure 1b.** Provided as an Excel file.

File Name: Supplementary Data 3
Description: **Related to Figure 1. List of the genes differentially expressed in *Pngl*$^{-/-}$ midguts that exhibited >1.5 fold-change in all three pair-wise comparisons based on RNA-seq.** Provided as an Excel file.

File Name: Supplementary Data 4
Description: **Related to Figure 1. List of the top 15 biological processes identified upon GO analysis of the upregulated and downregulated gene categories based on DAVID analysis.** Analysis of differentially expressed genes is based on the *P* value and false discovery rate (FDR) calculations embedded in the DAVID website. The gene count, *P* value, fold enrichment, FDR, and the gene list for each category is provided as an Excel file.
