# Supplementary material for: Gut barrier defects, intestinal immune hyperactivation and enhanced lipid catabolism drive lethality in NGLY1-deficient Drosophila
Source: Nat Commun. 2023 Sep 13;14:5667. doi: 10.1038/s41467-023-40910-w (PMC10499810; doi:10.1038/s41467-023-40910-w)
Supplement: Supplementary file 8 — Reporting Summary [file 41467_2023_40910_MOESM8_ESM.pdf]

nature portfolio

Corresponding author(s): Hamed Jafar-Nejad

Last updated by author(s): Jul 31, 2023

Reporting Summary

Nature Portfolio wishes to improve the reproducibility of the work that we publish. This form provides structure for consistency and transparency in reporting. For further information on Nature Portfolio policies, see our Editorial Policies and the Editorial Policy Checklist.

Statistics

For all statistical analyses, confirm that the following items are present in the figure legend, table legend, main text, or Methods section.

n/a	Confirmed	
☐	☒	The exact sample size (*n*) for each experimental group/condition, given as a discrete number and unit of measurement
☐	☒	A statement on whether measurements were taken from distinct samples or whether the same sample was measured repeatedly
☐	☒	The statistical test(s) used AND whether they are one- or two-sided<br>*Only common tests should be described solely by name; describe more complex techniques in the Methods section.*
☒	☐	A description of all covariates tested
☐	☒	A description of any assumptions or corrections, such as tests of normality and adjustment for multiple comparisons
☐	☒	A full description of the statistical parameters including central tendency (e.g. means) or other basic estimates (e.g. regression coefficient) AND variation (e.g. standard deviation) or associated estimates of uncertainty (e.g. confidence intervals)
☐	☒	For null hypothesis testing, the test statistic (e.g. *F*, *t*, *r*) with confidence intervals, effect sizes, degrees of freedom and *P* value noted<br>*Give P values as exact values whenever suitable.*
☒	☐	For Bayesian analysis, information on the choice of priors and Markov chain Monte Carlo settings
☒	☐	For hierarchical and complex designs, identification of the appropriate level for tests and full reporting of outcomes
☒	☐	Estimates of effect sizes (e.g. Cohen's *d*, Pearson's *r*), indicating how they were calculated

Our web collection on statistics for biologists contains articles on many of the points above.

Software and code

Policy information about availability of computer code

Data collection	Leica TCS SP8 confocal microscope and LAS X 3.1.5 microscope software were used for image acquisition.
Data analysis	GraphPad Prism 9 was used for statistical analysis. AMIRA 5.2.2 and ImageJ1.47 were used for image processing and quantification. cutadapt 1.12 (https://cutadapt.readthedocs.io/en/stable), edgeR 3.30.3 (https://bioconductor.org/packages/release/bioc/html/edgeR.html), R 3.65 (https://www.r-project.org), STAR 2.5.3a (https://github.com/alexdobin/STAR), Vennerable 3.1.0.9000 (https://github.com/js229/Vennerable) and shiny 1.6.0 (https://www.rstudio.com/products/shiny) were used for differential gene expression (DGE) analysis.

For manuscripts utilizing custom algorithms or software that are central to the research but not yet described in published literature, software must be made available to editors and reviewers. We strongly encourage code deposition in a community repository (e.g. GitHub). See the Nature Portfolio guidelines for submitting code & software for further information.

Data

Policy information about availability of data

All manuscripts must include a data availability statement. This statement should provide the following information, where applicable:

- Accession codes, unique identifiers, or web links for publicly available datasets
- A description of any restrictions on data availability
- For clinical datasets or third party data, please ensure that the statement adheres to our policy

Data are available within the article and supplementary information. Source data are provided as Source Data file with this paper. RNA-seq data generated in this study are available in the National Center for Biotechnology Information Gene Expression Omnibus database under the accession number GSE206229 (https://ncbi.nlm.nih.gov/geo/query/acc.cgi?acc=GSE206229).

Human research participants

Policy information about studies involving human research participants and Sex and Gender in Research.

Reporting on sex and gender	n/a
Population characteristics	n/a
Recruitment	n/a
Ethics oversight	n/a

Note that full information on the approval of the study protocol must also be provided in the manuscript.

Field-specific reporting

Please select the one below that is the best fit for your research. If you are not sure, read the appropriate sections before making your selection.

☒ Life sciences ☐ Behavioural & social sciences ☐ Ecological, evolutionary & environmental sciences

For a reference copy of the document with all sections, see nature.com/documents/nr-reporting-summary-flat.pdf

Life sciences study design

All studies must disclose on these points even when the disclosure is negative.

Sample size	Sample size was chosen to assure significant statistical differences and the reproducibility of the results. No sample size calculation was performed. For quantitative experiments, the number of animals per group were determined based on similar previous studies from our lab and others using similar methodologies (PMID: 33315951; 32720893; 21533227; 28930984). Each experiment was repeated at least three times. Detailed sample size information is provided in the figure legends.
Data exclusions	No data were excluded in the analysis.
Replication	All experiments were performed at least in three independent replicates with comparable results in all attempts. For the RNAseq experiments only two replicates were used per genotype and both replicates showed comparable results.
Randomization	In all experiments, animals per group were randomly allocated.
Blinding	Blinding was not required for the experiments. In all experiments, sample preparation, data acquisition and analysis were performed using the same conditions for all groups/samples regardless of their identity.

Reporting for specific materials, systems and methods

We require information from authors about some types of materials, experimental systems and methods used in many studies. Here, indicate whether each material, system or method listed is relevant to your study. If you are not sure if a list item applies to your research, read the appropriate section before selecting a response.

Materials & experimental systems

n/a	Involved in the study
☐	☒ Antibodies
☒	☐ Eukaryotic cell lines
☒	☐ Palaeontology and archaeology
☐	☒ Animals and other organisms
☒	☐ Clinical data
☒	☐ Dual use research of concern

Methods

n/a	Involved in the study
☒	☐ ChIP-seq
☒	☐ Flow cytometry
☒	☐ MRI-based neuroimaging

Antibodies

Antibodies used

Rabbit anti-dFoxo 1:250 (Abcam ab195977), Rabbit anti-pFoxo1 1:1000 (CST, Cat#9461), Rabbit anti-Akt 1:1000 (CST, Cat#4691), Rabbit anti-pAkt 1:1000 (CST Cat#4060), Mouse anti-β-Actin 1:1000 (DSHB Cat#224236), Rabbit anti-SAPK/JNK (Sigma Cat#559309), Helix pomatia agglutinin (HPA), Alexa FluorTM 488 conjugate 1:1000 (Invitrogen Cat#L11271), Wheat germ agglutinin (WGA) CF®488A 1:1000 (Biotium Cat#29022), Concanavalin A (ConA) CF®488A 1:1000 (Biotium Cat#29016)

Validation

All the antibodies used in the study are commercially available and validated by the the manufacturers. Specific validation information for each primary antibody is available on the manufacturer's website.
Rabbit anti-dFoxo 1:250 (Abcam ab195977), https://www.abcam.com/products/primary-antibodies/forkhead-box-protein-odfoxo-antibody-n-terminal-ab195977.html
Rabbit anti-pFoxo1 1:1000 (CST, Cat#9461), https://www.cellsignal.com/product/productDetail.jsp?productId=9461
Rabbit anti-Akt 1:1000 (CST, Cat#4691), https://www.cellsignal.com/product/productDetail.jsp?productId=4691
Rabbit anti-pAkt 1:1000 (CST Cat#4060), https://www.cellsignal.com/products/primary-antibodies/phospho-akt-ser473-d9e-xp-rabbit-mab/4060
Mouse anti-β-Actin 1:1000 (DSHB Cat#JLA20), https://dshb.biology.uiowa.edu/JLA20
Rabbit anti-SAPK/JNK (Millipore Sigma Cat#559309),https://www.emdmillipore.com/US/en/products/

Animals and other research organisms

Policy information about studies involving animals; ARRIVE guidelines recommended for reporting animal research, and Sex and Gender in Research

Laboratory animals

Species: Drosophila melanogaster
Strains: Wild-type, mutant and transgenic fly strains were either obtained from Bloomington Stock center or previously generated in our lab or requested from other investigators.
(1) y w (BDSC # 6598)
(2) foxoΔ94/TM6B, Tb1 (BDSC # 42220)
(3) Tl/TM3, Sb1 (BDSC # 3238)
(4) RelE38 (BDSC # 9458)
(5) UAS-foxo RNAi (BDSC # 80950)
(6) UAS-Tl RNAi (BDSC # 31044)
(7) UAS-Rel RNAi (BDSC # 33661)
(8) Myo1A-GAL4 (BDSC # 83278)
(9) r4-GAL4 (BDSC # 33832)
(10) c135-GAL4 (path-GAL4) (BDSC # 6978)
(11) UAS-dipl2 (BDSC # 80936)
(12) UAS-InRWT (BDSC # 8262)
(13) UAS-InRA1325D (BDSC # 8263)
(14) UAS-AktWT (BDSC # 8192)
(15) UAS-AktΔPH ((BDSC # 80935)
(16) UAS-mCherry nls (BDSC # 38424)
(17) pros-GAL4 (BDSC # 84276)
(18) esg-GAL4 (BDSC # 93857)
(19) elav-GAL4 (BDSC # 99269)
(20) C147-GAL4 (BDSC # 6979)
(21) UAS-bsk RNAi (BDSC # 53310)
(22) Mef2-GAL4 (Brand & Perrimon, 1993; PMID: 8223268)
(23) Pnglex14 (Funakoshi et al., 2010; PMID: 20479940)
(24) UAS-PnglWT (Funakoshi et al., 2010; PMID: 20479940)
(25) UAS-PnglC303A (Funakoshi et al., 2010; PMID: 20479940)
(26) PBac{Pnglwt}VK31 (Pngl duplication; Han et al., 2020; PMID: 33315951)
(27) Dp(1;3)DC102, PBac{DC102}VK33 (AMPKα duplication; Venken et al., 2010; PMID: 20876565)
(28) UAS-dipl6 (Slaidina et al., 2009; PMID: 20059956)

Age: Third instar larvae were used in this study. For lethality assay experiment, adult flies of 3-5 days were used to set up crosses. Specific age in terms of hours after egg laying (AEL) is mentioned in the results and Materials and Methods section.

Wild animals

No wild animals were used in the study.

Reporting on sex	Sex were not separated for the study. Both male and female flies were used for the study. Information is mentioned in the manuscript.
Field-collected samples	Study did not involved sample collection from field.
Ethics oversight	No ethic approval was required.

Note that full information on the approval of the study protocol must also be provided in the manuscript.
